# Case Report: Reopening the anabolic window — romosozumab added to ongoing denosumab for severe osteoporosis in two kidney transplant recipients

**DOI:** 10.3389/fendo.2026.1947545

**Published:** 2026-09-04

**Authors:** Simona Barbuto, Paolo Mastromauro, Guido Zavatta, Daniele Vetrano, Anna Mistretta, Nicolò Bisceglia, Giorgia Comai, Uberto Pagotto, Gaetano La Manna, Giuseppe Cianciolo

**Affiliations:** 1 Nephrology, Dialysis and Kidney Transplant Unit, IRCCS Azienda Ospedaliero-Universitaria di Bologna, Bologna, Italy; 2 Department of Medical and Surgical Science (DIMEC), Alma Mater Studiorum - University of Bologna, Bologna, Italy; 3 Division of Endocrinology and Diabetes Prevention and Care, IRCCS Azienda Ospedaliero-Universitaria di Bologna, Bologna, Italy

**Keywords:** antifracture therapy, bone mineral densisty, CKD-MBD (chronic kidney disease - mineral bone disorder), combination therapy, denosumab (anti-RANKL antibody), kidney transplant, osteoporosis, romosozumab (anti-sclerostin antibody)

## Abstract

Kidney transplant recipients develop disorders of mineral and bone metabolism that further impair bone strength, leading to osteoporosis and a high fracture risk. However, evidence in this setting is limited. Romosozumab, a sclerostin inhibitor with both anabolic and antiresorptive effects, is an attractive option, but its use in transplant recipients, as well as its combination with denosumab, has rarely been reported. We describe two female kidney transplant recipients with severe osteoporosis and persistently high fracture risk despite long-term denosumab, in whom romosozumab (210 mg monthly) was added to ongoing denosumab for 12 months. In both patients the bone-formation markers P1NP and BAP rose early, and bone mineral density improved preferentially at the lumbar spine (Case 1 + 5.9%, Case 2 + 11.2%), with only modest changes at the femoral neck and total hip. Serum calcium, parathyroid hormone, 25-hydroxyvitamin D and allograft function remained stable throughout, with no clinically significant hypocalcemia, and neither patient sustained a new vertebral fracture. Adding romosozumab to ongoing denosumab thus reopened the anabolic window and improved lumbar-spine bone mineral density without compromising mineral metabolism or graft function. This combination may represent a reasonable option for carefully selected transplant recipients who continue to lose bone or to fracture despite antiresorptive therapy, pending confirmation in controlled studies.

## Introduction

Kidney transplant recipients (KTRs) have an elevated risk of bone loss and fragility fractures, driven by a confluence of factors: pre-existing chronic kidney disease–mineral and bone disorder (CKD-MBD), chronic immunosuppressive therapy, diabetes mellitus (whether or not related to corticosteroid therapy), and the progressive decline of allograft function (1). This condition is recently defined as CKD-associated osteoporosis. Some studies have documented a reduction in bone mineral density (BMD) of up to 10% within the first 6 months post-transplant, with an additional 0.4–4.5% reduction between 6 and 12 months (2). These changes translate into a clinically meaningful burden: 22.5% of kidney transplant recipients sustain at least one fracture within 5 years of transplantation, a rate roughly four times that of the general population (3).

From a pathophysiological standpoint, bone turnover declines broadly over the first post-transplant year, a shift often accompanied by stabilization or even modest recovery of bone mass. In a meaningful subset of KTRs, however, this decline of turnover is excessive, tipping the balance toward adynamic bone disease and net bone loss (4, 5). Jørgensen et al. identified persistent post-transplant hypophosphatemia and low pre-transplant bone turnover as important independent contributors to this process, alongside the well-recognized effects of corticosteroids and immunosuppression (4).

The last Kidney Disease Improving Global Outcomes (KDIGO) guidelines for the management of CKD-associated osteoporosis in KTRs provide general recommendations; however, the scientific evidence supporting antiosteoporotic therapies remains limited, and therapeutic decisions are therefore based on clinical experience (2, 6). Calcium and vitamin D supplementation form the cornerstone of supportive therapy (7, 8). Because vitamin D deficiency promotes hypocalcemia and subsequent bone loss, the KDIGO guidelines recommend correcting vitamin D deficiency and insufficiency in KTRs using the treatment strategies used in the general population (6, 9, 10).

When overt osteoporosis occurs alongside high bone turnover, antiresorptive therapy, including bisphosphonates and denosumab, is reasonable. Bisphosphonates have demonstrated efficacy in maintaining or improving BMD at the lumbar spine and femoral neck in the early post-transplant setting (11, 12) and may also reduce fracture risk (13). Their nephrotoxic potential, however, necessitates caution, and they are generally avoided when eGFR falls below 30 mL/min (14). Denosumab is a fully human anti-RANKL monoclonal antibody indicated for the treatment of osteoporosis and fracture prevention. By inhibiting osteoclast development and activity, denosumab reduces bone resorption and increases BMD (15). Likewise, among *de novo* KTRs on corticosteroid-containing immunosuppressive regimens, its use has been associated with significant gains in BMD at both axial and appendicular skeletal sites (16).

Currently approved therapies for the treatment of postmenopausal osteoporosis in the general population include anabolic agents — teriparatide and abaloparatide — as well as romosozumab, a monoclonal antibody targeting sclerostin that exerts dual actions on both bone formation and bone resorption (17).

The availability of these agents has expanded the therapeutic options for patients in whom antiosteoporotic therapy does not achieve the expected outcomes, enabling sequential and combination treatment strategies (18, 19). Antiosteoporotic therapy should therefore be tailored to the underlying bone phenotype and bone turnover state, assessed at treatment initiation and during follow-up (20).

Further deterioration of BMD or the occurrence of new low-trauma fractures despite ongoing antiresorptive therapy is not uncommon in KTRs. As in the general population, this clinical scenario warrants consideration of sequential or combination approaches; nevertheless, the evidence is limited.

Herein, we report two female KTRs with severe osteoporosis and persistent high fracture risk despite denosumab, in whom romosozumab was added to ongoing denosumab as a combination regimen over a 12-month period.

## Case description

### Case 1

A 78-year-old woman started combination therapy with denosumab and romosozumab in January 2025. Chronic kidney disease was first documented in 1990, at the age of 43, in the context of a hypertensive crisis. At the age of 59, she underwent deceased-donor kidney transplantation after 10 months of peritoneal dialysis. Induction immunosuppression consisted of basiliximab, followed by maintenance therapy with tacrolimus, mycophenolate mofetil, and low-dose corticosteroids. Serum creatinine at discharge was 1.28 mg/dL. Before transplantation, treatment for CKD-MBD included calcitriol and sevelamer. Following transplantation, progressive deterioration of BMD prompted initiation of antiresorptive therapy with risedronate in 2008.

In October 2020, given severe osteoporosis (T-scores: lumbar spine –5.3, total hip –3.5, femoral neck –3.7) and the presence of three vertebral fractures (D6, D7, and D8), therapy was transitioned to denosumab 60 mg every 6 months, alongside supplementation with cholecalciferol 6125 IU weekly.

Denosumab was well tolerated; however, an early reduction in serum calcium with a subsequent rise in PTH prompted supplementation with calcitriol and calcium. Subsequent morphometric assessments in September 2022 and September 2023 confirmed stability of the previously identified deformities, with no new vertebral fractures throughout the entire denosumab monotherapy period.

Over the course of denosumab therapy, BMD showed meaningful gains at the lumbar spine and femoral neck. Compared with the pre-treatment DXA performed in 2020, lumbar spine BMD increased from 0.466 to 0.730 g/cm² (+56.7%, T-score from –5.3 to –3.8) and femoral neck BMD from 0.442 to 0.569 g/cm² (+28.7%, T-score from –3.7 to –3.4) by September 2024. Total hip BMD showed more modest change, from 0.513 to 0.537 g/cm² (+4.7%, T-score from –3.5 to –3.3). Despite these gains, T-scores at all sites remained in the severe osteoporosis range at the end of the denosumab monotherapy period. The 10-year probability of major osteoporotic fracture estimated by the FRAX algorithm (Italy, BMD-adjusted) was 34%, with a hip fracture probability of 11%; these estimates confirmed a persistently high residual fracture risk.

After 4 years of denosumab therapy, in light of severe persistent osteoporosis, and following exclusion of a history of myocardial infarction and stroke, combination treatment with denosumab and romosozumab 210 mg monthly (administered as two subcutaneous injections of 105 mg each) was initiated in January 2025, continuing the existing cholecalciferol and calcitriol supplementation.

#### Bone mineral density

**Figure 1:**
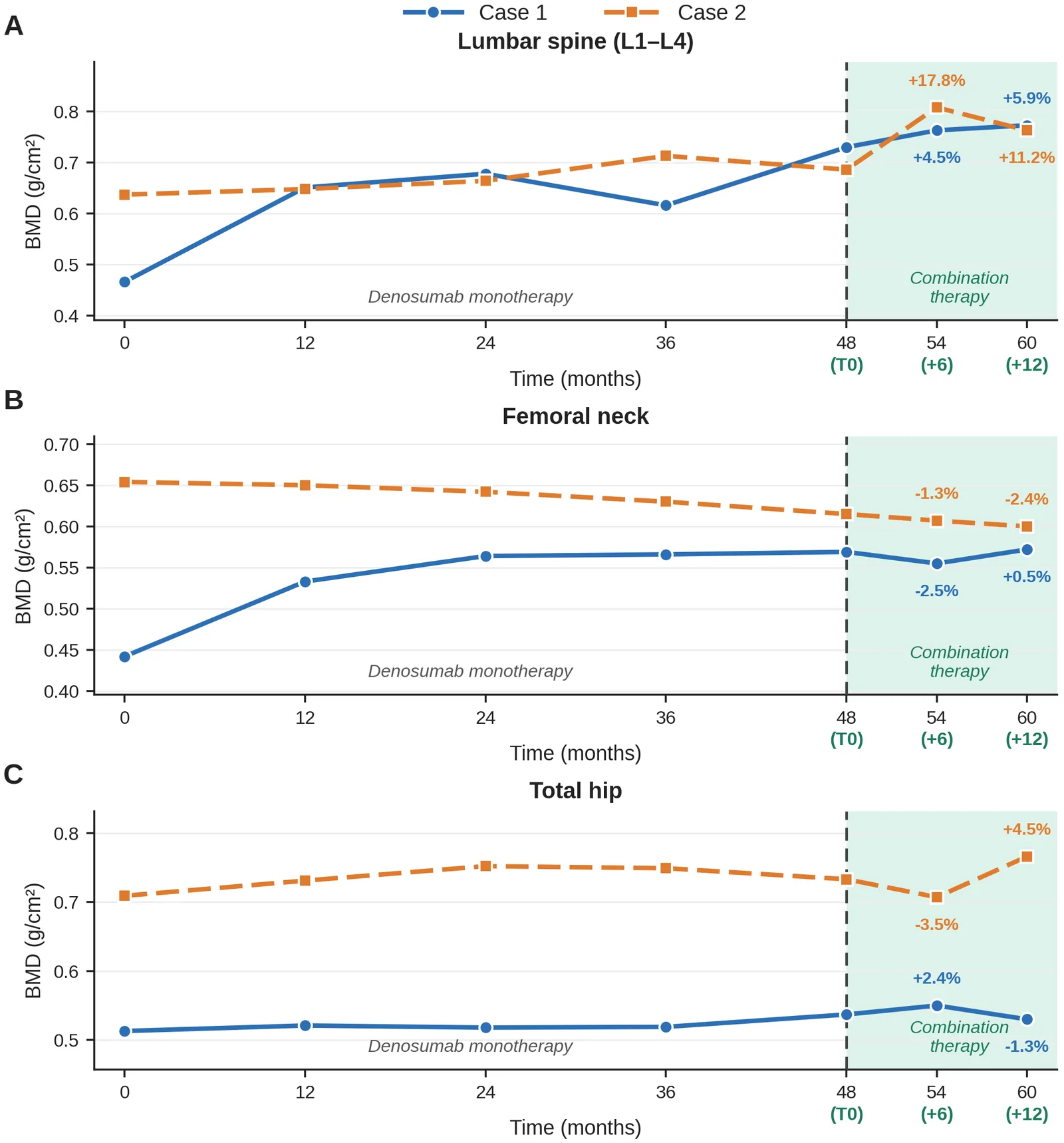
Bone mineral density (BMD) at the **(A)** lumbar spine (L1–L4), **(B)** femoral neck, and **(C)** total hip in Case 1 (blue circles) and Case 2 (orange squares), spanning four years of denosumab monotherapy (white background) and the subsequent 12 months of combination therapy with denosumab and romosozumab (shaded background). The dashed vertical line marks the initiation of combination therapy, which corresponds to month 48 of denosumab monotherapy and to the baseline (T0) of the combined regimen. Labels indicate the percentage change in BMD relative to T0.

**Figures 1A–C** shows the percentage change in BMD at the lumbar spine, femoral neck, and total hip during combination therapy. In detail, the most recent DXA prior to treatment initiation demonstrated severe osteoporosis at all skeletal sites: lumbar spine (L1–L4) BMD 0.730 g/cm² (T-score –3.8), femoral neck BMD 0.569 g/cm² (T-score –3.4), and total hip BMD 0.537 g/cm² (T-score –3.3). TBS was partially degraded (1.279). At the end of combination therapy, lumbar spine BMD was 0.773 g/cm² (+5.9% vs. baseline, T-score –3.4), femoral neck BMD 0.572 g/cm² (+0.5% vs. baseline, T-score –3.4), and total hip BMD 0.530 g/cm² (–1.3% vs. baseline, T-score –3.4). TBS remained partially degraded (1.243). Vertebral morphometry performed at the end of combination therapy demonstrated stability of the pre-existing vertebral deformities compared with baseline, with no new vertebral fractures identified.

#### Bone turnover markers

**Figure 2:**
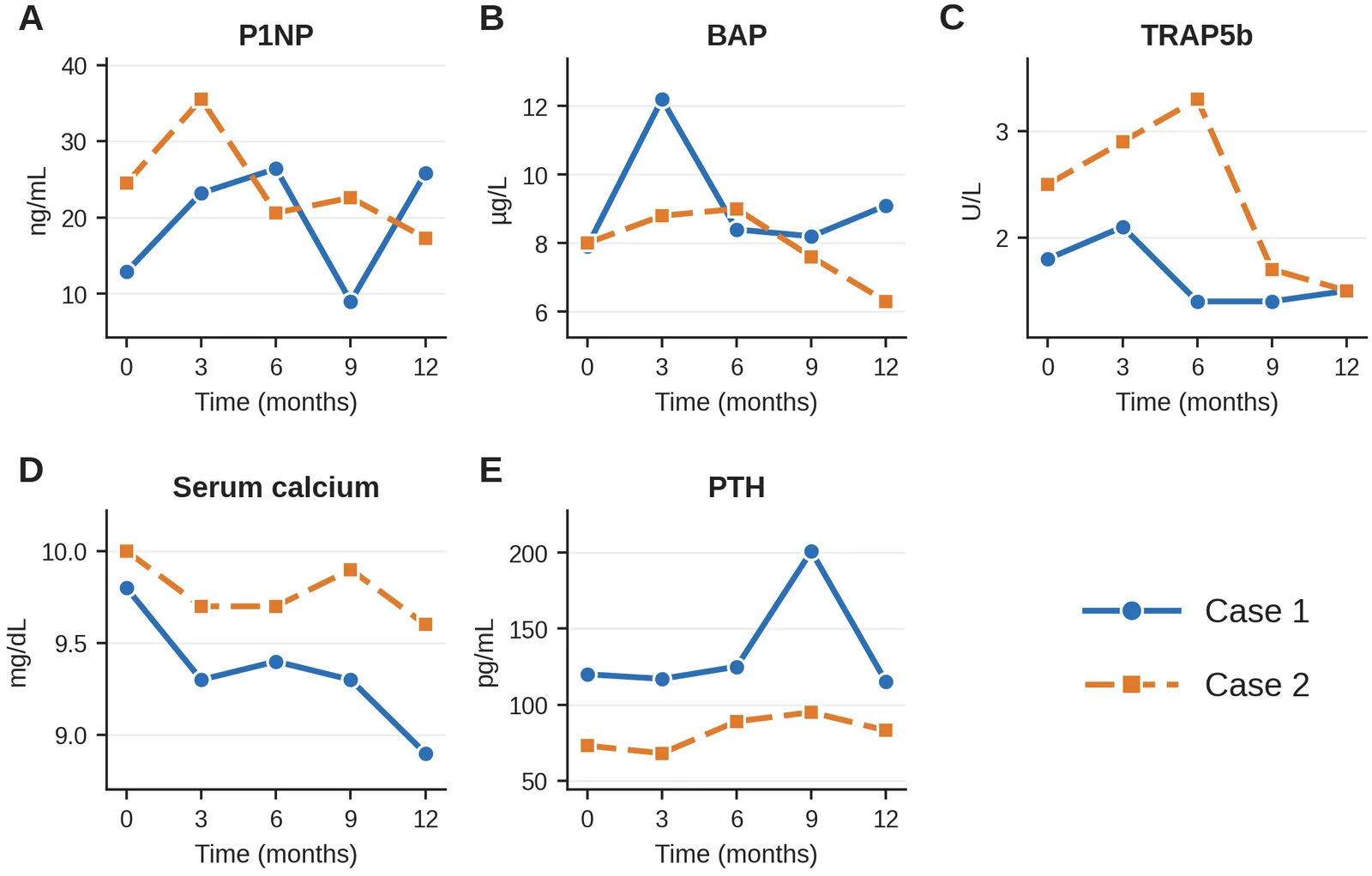
Time course of bone turnover markers and mineral metabolism parameters during 12 months of combination therapy with denosumab and romosozumab in Case 1 (blue circles) and Case 2 (orange squares). **(A)** Procollagen type I N-terminal propeptide (P1NP); **(B)** bone alkaline phosphatase (BAP); **(C)** tartrate-resistant acid phosphatase 5b (TRAP5b); **(D)** serum calcium; **(E)** parathyroid hormone (PTH). Measurements were obtained at baseline (month 0) and at 3, 6, 9, and 12 months after initiation of combination therapy. The bone formation markers P1NP and BAP rose early in both patients, while TRAP5b showed a transient increase followed by a progressive decline; serum calcium remained substantially stable throughout follow-up. PTH showed a transient peak at month 9 in Case 1, whereas it remained stable in Case 2.

**Figures 2A–C** shows the time course of P1NP, BAP, and TRAP5b during the combination therapy. The bone formation markers P1NP and BAP increased rapidly during the first months of combination therapy, whereas TRAP5b showed a transient increase in the third month after treatment initiation, followed by a progressive decline, remaining below baseline thereafter. P1NP transiently declined to 9.0 ng/mL at month 9 before recovering to 25.8 ng/mL at month 12.

#### Calcium, phosphate, PTH and vitamin D

**Figures 2D, E** shows the time course of serum calcium and PTH during combination therapy. Serum calcium remained stable throughout the combination period, ranging from 8.9 to 9.8 mg/dL, with no episodes of clinically significant hypocalcemia. Serum phosphate was persistently low-normal, fluctuating between 1.9 and 2.7 mg/dL. PTH levels showed notable variability, peaking at 201 pg/mL at 9 months, with no apparent association with hypocalcemia or worsening allograft function. Vitamin D 25-OH levels remained stable throughout the follow-up, ranging from 30 to 37 ng/mL at all available timepoints.

#### Renal function

Allograft function remained stable throughout combination therapy. Creatinine was 1.19 mg/dL (eGFR 45 mL/min) at the start of therapy and 0.91 mg/dL (eGFR 60 mL/min) at the end.

### Case 2

A 74-year-old woman initiated combination therapy with denosumab and romosozumab in January 2025. She had been diagnosed with chronic kidney disease secondary to scleroderma renal crisis at the age of 63, requiring renal replacement therapy for approximately 3 years. At the age of 66, she underwent deceased-donor kidney transplantation. At discharge, serum creatinine was 1.16 mg/dL, and maintenance immunosuppressive therapy with tacrolimus, mycophenolic acid, and low-dose corticosteroids was prescribed.

Osteoporosis with multiple vertebral fractures was diagnosed at the age of 69. Denosumab 60 mg every 6 months, alongside weekly cholecalciferol supplementation, was started in January 2021.

During denosumab monotherapy, compared with the pre-treatment DXA performed in October 2020, lumbar spine BMD increased from 0.637 to 0.686 g/cm² (+7.7%, T-score from –4.5 to –4.1) and total hip BMD from 0.709 to 0.733 g/cm² (+3.4%, T-score from –2.4 to –2.2) by October 2024. In contrast, femoral neck BMD declined from 0.654 to 0.615 g/cm² (–6.0%, T-score from –2.7 to –3.0). Despite the modest densitometric gains, severe osteoporosis persisted. The 10-year probability of major osteoporotic fracture estimated by the FRAX algorithm (Italy, BMD-adjusted) was 42%, with a hip fracture probability of 10%. Moreover, vertebral morphometry demonstrated progressive worsening of the fracture burden during this period, with the development of three new vertebral fractures. In November 2024, the patient also sustained a left wrist fracture.

In light of this progressive deterioration despite ongoing antiresorptive therapy and following exclusion of a history of myocardial infarction and stroke, combination treatment with denosumab and romosozumab 210 mg monthly (administered as two subcutaneous injections of 105 mg each), alongside weekly cholecalciferol supplementation at a dose of 12, 500 IU, was initiated in January 2025, following approval by the institutional drug committee.

#### Bone mineral density

**Figures 1A–C** shows the percentage change in BMD at the lumbar spine, femoral neck, and total hip during combination therapy. In detail, the most recent DXA prior to treatment initiation confirmed lumbar severe osteoporosis: L1–L4 BMD 0.686 g/cm² (T-score –4.1), femoral neck BMD 0.615 g/cm² (T-score –3.0), and total hip BMD 0.733 g/cm² (T-score –2.2). TBS was partially degraded (1.191). At the end of combination therapy, L1–L4 BMD was 0.763 g/cm² (+11.2% vs. baseline, T-score –2.6), femoral neck BMD 0.600 g/cm² (–2.4% vs. baseline, T-score –3.2), and total hip BMD 0.766 g/cm² (+4.5% vs. baseline, T-score –1.5). Vertebral morphometry performed at the end of combination therapy demonstrated stability of the multiple pre-existing vertebral deformities compared with baseline, with no new vertebral fractures identified.

#### Bone turnover markers

**Figures 2A–C** shows the time course of P1NP, BAP, and TRAP5b during combination therapy. As already observed in Case 1, the bone formation markers P1NP and BAP increased rapidly during the first months of combination therapy. TRAP5b again showed a transient increase peaking at the sixth month, followed by a progressive decline.

#### Calcium, phosphate, PTH, and vitamin D

**Figures 2D, E** shows the time course of serum calcium and PTH during combination therapy. Serum calcium remained within the normal range throughout the combination period, ranging from 9.6 to 10.0 mg/dL, with no episodes of clinically significant hypocalcemia and no meaningful variation at any time point. Serum phosphate remained stable during combination therapy, ranging from 3.2 to 3.7 mg/dL. PTH showed stability throughout the follow-up. Vitamin D 25-OH levels were suboptimal at baseline (27 ng/mL), recovering to 35 ng/mL at the end of combination therapy.

#### Renal function

Allograft function remained aroud stable throughout combination therapy. Creatinine was 1.33 mg/dL (eGFR 41 mL/min) at the start of therapy and 1.92 mg/dL (eGFR 25 mL/min) at the end. Case 1 and two are summarized in **Table 1**.

**Table 1:** Bone mineral density (lumbar spine, femoral neck, total hip) and clinical course across therapy phases in the two kidney transplant recipients. BMD is given in g/cm² with, in parentheses, the T-score and — from the second timepoint — the percentage change versus the preceding phase, shown in each cell from top to bottom: baseline (before denosumab) → after denosumab monotherapy (immediately before adding romosozumab) → after 12 months of combination therapy. q6mo, every 6 months.

*Parameter*	Case 1	Case 2
Prior an*tire*sorptive	Risedronate (2008)	None
Denosumab (start; regimen)	Oct 2020; 60 mg q6mo	Jan 2021; 60 mg q6mo
Romosozumab added (start; regimen)	Jan 2025; 210 mg monthly	Jan 2025; 210 mg monthly
	*Bone mineral density, g/cm² (T-score; % change)*	
Lumbar spine (L1–L4)	Baseline: 0.466 (−5.3) After denosumab: 0.730 (−3.8; +56.7%) After combination: 0.773 (−3.4; +5.9%)	Baseline: 0.637 (−4.5) After denosumab: 0.686 (−4.1; +7.7%) After combination: 0.763 (−2.6; +11.2%)
Femoral neck	Baseline: 0.442 (−3.7) After denosumab: 0.569 (−3.4; +28.7%) After combination: 0.572 (−3.4; +0.5%)	Baseline: 0.654 (−2.7) After denosumab: 0.615 (−3.0; −6.0%) After combination: 0.600 (−3.2; −2.4%)
Total hip	Baseline: 0.513 (−3.5) After denosumab: 0.537 (−3.3; +4.7%) After combination: 0.530 (−3.4; −1.3%)	Baseline: 0.709 (−2.4) After denosumab: 0.733 (−2.2; +3.4%) After combination: 0.766 (−1.5; +4.5%)
New vertebral fractures (denosumab/combination)	0/0	3/0 (+ wrist, 2024)

## Discussion

Bone disease in KTRs is a common condition following kidney transplantation. It reflects the CKD-MBD inherited from the pre-transplant period, the skeletal toxicity of corticosteroids and calcineurin inhibitors, and the ongoing decline in allograft function; the resulting bone phenotype is varied and cannot be reliably predicted by kidney function alone (1, 2, 4, 5). The fracture burden is correspondingly high, with roughly 1 in 4 recipients fracturing within 5 years (3). Yet the evidence base for antiosteoporotic therapy in KTRs is slight, and KDIGO guidelines only suggest (2, 6, 20): calcium and vitamin D repletion (7, 8), bisphosphonates to preserve BMD early after transplantation but are limited once eGFR falls below 30 mL/min (9, 11–13), and denosumab offers a non-nephrotoxic antiresorptive alternative (15, 16). A distinct and demanding scenario is the patient who continues to lose bone or to fracture despite adequate antiresorptive therapy — a situation that, as in the general population, warrants anabolic or combination strategies (18, 19, 21) but has scarcely been explored after transplantation, and that motivated the treatment of the two recipients reported here.

Denosumab, a fully human anti-RANKL antibody, produces sustained BMD gains and fracture reduction in postmenopausal osteoporosis and, being non-renally cleared, is attractive across the spectrum of kidney function (15); in *de novo* KTRs the randomized trial by Bonani et al. showed it prevented post-transplant bone loss (16). Its strong inhibition of resorption, however, increases the frequency of early hypocalcemia and a reactive PTH rise in this population (15), as demonstrated in Case 1, where the expected decline in calcium was effectively managed with calcitriol and calcium — with a PTH response that is best understood as physiological and an indirect indicator of efficacy rather than a complication (15). Both patients gained lumbar-spine BMD on denosumab (Case 1 + 56.7%, Case 2 + 7.7%), yet both remained within the severe osteoporosis range, with FRAX 10-year major-fracture probabilities of 34% and 42%, and Case 2 continued to fracture (three new vertebral deformities and a wrist fracture) despite continual treatment. This illustrates the limitations of a purely antiresorptive strategy: denosumab delivers its largest increments early and then plateaus, exerts only limited, mostly modeling-based bone-forming activity (20), and cannot be simply withdrawn because discontinuation provokes rebound vertebral fractures (18). Persistent severe osteoporosis and ongoing fractures thus reflect a high residual fracture risk despite denosumab and justify adding an osteoanabolic agent rather than continuing or switching antiresorptive treatment. Combination therapy is based on the premise that concomitant use of anabolic and antiresorptive agents may confer greater benefit than monotherapy, particularly in patients with persistent fractures despite treatment (20).

Romosozumab, a monoclonal antibody against sclerostin, is unique in acting on both arms of turnover: by de-repressing Wnt signaling it stimulates osteoblastic bone formation while limiting resorption, opening a transient anabolic window that yields rapid BMD gains, most marked at the trabecular-rich lumbar spine (17), and it reduced fractures in the pivotal trials against both placebo (FRAME) and alendronate (ARCH) (17, 22). This modeling-based formation is exactly what a patient plateaued on denosumab lacks. Evidence in kidney disease is limited but reassuring at moderate stages: a *post-hoc* analysis of FRAME and ARCH found romosozumab effective and well tolerated down to an eGFR of approximately 30 mL/min, with fracture reduction preserved across kidney-function strata and no adverse effect on renal function (23). Patients with severe CKD, on dialysis, or with a functioning allograft were not represented, however, and sclerostin’s vascular role raises an unresolved concern about aggravating vascular calcification in the uremic milieu. Two safety considerations shaped our practice: abrupt stimulation of remodelling can trigger hypocalcemia, so we recommend calcium and vitamin D repletion.

Because sclerostin also participates in the bone–vascular axis, its inhibition raises the theoretical concern that it could aggravate vascular calcification in the uremic milieu (20). An excess of serious cardiovascular events was seen with romosozumab versus alendronate in ARCH, though not versus placebo in FRAME, so the drug is contraindicated after recent myocardial infarction or stroke (22). Accordingly, in both recipients, treatment began only after excluding prior myocardial infarction and stroke and optimising calcium and vitamin D.

The concern when adding an anabolic agent to long-term denosumab is that deep, continuous remodelling suppression might blunt the response. Adami et al. addressed this directly: in women on denosumab for more than two years, adding romosozumab still produced a rapid P1NP rise and a significant lumbar-spine BMD gain (+7.2%), though not at the femoral neck, showing that romosozumab retains its anabolic potential over a denosumab background — the anabolic window can be reopened (19, 22, 24). Comparable gains from adding romosozumab to ongoing denosumab have since been reported in other combination cohorts (25, 26). Notably, whereas romosozumab alone causes a transient calcium fall with a compensatory PTH rise, calcium and PTH remained stable during the combination, because on denosumab osteoclast-mediated calcium efflux is already maximally suppressed (23, 24).

Our two KTRs summarise this pattern. In both, P1NP and BAP rise early after romosozumab was added and BMD improved preferentially at the lumbar spine (Case 1 + 5.9%, Case 2 + 11.2%), with only modest or neutral changes at the femoral neck and total hip — the same trabecular-dominant response reported by Adami (19) — while TRAP5b showed a transient increase then decline, consistent with a coupled but net-anabolic effect. That P1NP increased at all, despite the low-turnover state imposed by long-term denosumab, is notable, since baseline bone-formation activity, as reflected by PINP, is itself a determinant of the BMD gain achievable with romosozumab (27). Serum calcium remained stable with no clinically significant hypocalcemia, PTH remained largely stable (aside from a transient month-9 peak in Case 1 without hypocalcemia or graft dysfunction), vitamin D remained replete, and allograft function was unchanged over 12 months. Crucially, neither patient sustained a new vertebral fracture during combination therapy, in contrast to the fracture accrual seen in Case 2 on denosumab alone.

These observations must be interpreted cautiously. This is a report of only two patients over 12 months; the outcome was BMD rather than fracture; and the durability and, above all, the long-term cardiovascular and vascular safety of romosozumab in transplant recipients remain unproven. The optimal duration of the anabolic phase (28) and the best subsequent strategy — whether to continue denosumab alongside romosozumab or resume it afterwards, as in sequential romosozumab-to-denosumab regimens (29–31) — remain unresolved. Although romosozumab has recently been reported in kidney transplant recipients (32), combination osteoporosis therapy has not been systematically evaluated in CKD or transplant populations, so these cases address a recognized evidence gap.

Within these limits, adding romosozumab to ongoing denosumab reopened the anabolic window, improved lumbar-spine BMD and bone formation, and was not accompanied by hypocalcemia or deterioration of graft function; pending controlled data, it may be a reasonable option for carefully selected recipients who continue to lose bone or to fracture despite antiresorptive therapy, provided cardiovascular contraindications are excluded and mineral metabolism and graft function are closely monitored.

## Data Availability

The original contributions presented in the study are included in the article/supplementary material. Further inquiries can be directed to the corresponding author.
